# A Case of Cannabinoid Hyperemesis Syndrome and Acute Kidney Injury: A Review of the Literature

**DOI:** 10.7759/cureus.34350

**Published:** 2023-01-29

**Authors:** Shannay E Bellamy, Brian Loor, Maria Gutierrez-Castillo

**Affiliations:** 1 Internal Medicine, Jersey City Medical Center, Jersey City, USA

**Keywords:** cannabinoid hyperemesis syndrome, cannabinoid, nausea and vomiting, hyperemesis, cannabis use

## Abstract

Cannabis is a commonly used illicit drug worldwide with many noted adverse effects and medical properties. It has been previously used in medicine for the management of chemotherapy-induced nausea and vomiting. The chronic use of cannabis is well-known to be associated with psychological and cognitive adverse effects, however, cannabinoid hyperemesis syndrome is a less commonly recognized complication of chronic long-term use of cannabis, though it does not occur in the majority of chronic cannabis users. Here we present the case of a 42-year-old male who presented with classical clinical features of cannabinoid hyperemesis syndrome.

## Introduction

Cannabis had been reported as the most commonly grown and used illicit drug, with about 2.5% of the world population consuming cannabis each year [1]. In the United States it is estimated that the lifetime prevalence of cannabis use is about 42% to 46% [2]. Acute use of cannabis has been associated with euphoric mood, decreased anxiety, depression and psychomotor slowing, while long-term chronic use of cannabis is associated with cognitive impairment, exacerbation of psychotic symptoms in patients with schizophrenia, tracheal and bronchial injury, inflammation of the lung parenchyma and increased risk of chronic bronchitis [1,3]. Cannabis is well-known in the general public and amongst medical personnel for its anti-emetic properties, particularly in patients with chemotherapy-induced nausea and vomiting. Cannabinoid hyperemesis syndrome (CHS) is a reported complication of chronic cannabis use. The prevalence of CHS is uncertain, ranging from 0.1% to 35% in the literature [4,5]. Here we present a case of a 42-year-old male with long-term significant cannabis use who presented with multiple hospital visits for nausea and vomiting characteristic of CHS.

## Case presentation

We present the case of a 42-year-old male with no significant past medical history who presented to the emergency room with a one-day history of multiple episodes of nausea and vomiting. He was in his usual state of health until 4 am on the morning prior to presentation when he awoke with severe nausea, followed by recurrent episodes of non-bilious non-bloody vomiting and generalized cramping abdominal pain. He reports more that 12 episodes of vomiting prior to presenting to the emergency room, with one to two episodes occurring almost every hour. This patient also gave a history of brief improvement in nausea after having a warm shower on the morning of presentation as he was preparing for work, however, nausea and vomiting quickly returned and was unable to tolerate any oral intake including sips of water. He denied any diarrhea, constipation, abdominal distension, or fever. He did report the occurrence of epigastric burning pain radiating to the chest and throat following multiple episodes of vomiting. On the night prior to the onset of symptoms, he shared a meal with his family, however, no other family members had experienced similar symptoms. In his social history, he reported smoking daily marijuana use approximately 20 years, with an average use of 7-10g of marijuana weekly as reported by the patient, and he also admitted to have smoked marijuana the night prior to his presentation, quantified as "2 blunts". He denied any tobacco use or any other illicit drug use.

In addition, he gave a history of at least four prior emergency room visits for similar complaints over the past three years, with the most recent episode occurring four months prior. He reported smoking marijuana of varying quantities on the night prior to each episode. He recalls each episode occurring usually in the early mornings. He could was unsure if his symptoms has improved with any warm showers or baths on prior presentations. He denied any use of marijuana other illicit or prescribed drugs during the episodes of nausea and vomiting. He reported on each presentation, lab and imaging investigations revealed no etiology for his symptoms and his symptoms and he was diagnosed with acute gastritis and viral gastrointestinal illnesses. On each occasion he had resolution of his symptoms with 48 to 72 hours antiemetic therapy, during which time, he had no cannabis use. 

On this presentation, he had a blood pressure of 119/77mmHg, pulse rate of 80 beats per minute, respiratory rate of 18/min, temperature of 98.1 F and oxygen saturation of 98% on room air. His physical exam was significant for dry oral mucous membranes and generalized abdominal tenderness, more so in the periumbilical area, without any rebound tenderness or guarding. Examination of other systems was unremarkable. Laboratory investigations were significant for leukocytosis of 14.9x 103/mcL with a predominant neutrophilia, mild hyponatremia of 131mmol/L, hypochloremia of 94mmol/L, mild hypercalcemia of 11.7mg/dl, mild hyperglycemia of 130mg/dl and renal impairment with a blood urea nitrogen (BUN) of 28mg/dl and creatinine of 3.48mg/dl. On review of prior laboratory investigations done four months before, BUN and creatinine were both within normal limits. Blood glucose on presentation was 130mg/dl. Serum amylase and liver function tests were within normal limits (Table 1). Urine drug screen was positive for cannabis. Urinalysis was positive for ketones but otherwise was not suggestive of a urinary tract infection. Fractional excretion of sodium (FeNA) was 0.2%, suggestive of a pre-renal cause of renal impairment. Computerized tomography (CT) imaging of the abdomen and pelvis showed no acute intra-abdominal or pelvic pathology.

**Table 1:** Results of Initial Laboratory Investigations Hb - hemoglobin, MCV - mean corpuscular volume, WBC - white blood cell count, PT - prothrombin time, INR - international normalized ratio, PTT - activated partial thromboplastin time, Na - sodium, K - potassium, Cl - chloride, CO2 - serum bicarbonate, BUN - blood urea nitrogen, Ca - serum calcium, AST - aspartate aminotransferase, ALT - alanine aminotransferase, ALP - alkaline phosphatase

Laboratory test on admission	Result	Normal Range
Hb	17 g/dl	14-18g/dl
MCV	98 fl	80.0-100.0 fl
Platelets	306 K/UL	130 – 400K/UL
WBC	14.9 K/UL	4.5-11.0K/UL
PT	11.7 sec	12-15.1sec
INR	1.01	0.85-1.14
PTT	29.3 sec	25.4-36.7 sec
Na	131 mmol/L	136-145 mmol/L
K	4.7 mmol/L	3.5-5.1 mmol/L
Cl	94 mmol/L	98-107 mmol/L
CO2	26 mmol/L	20-31 mmol/L
BUN	28 mg/dl	9-23 mg/dl
Creatinine	3.48 mg/dl	0.70-1.30 mg/dl
Ca	11.7 mg/dl	8.7-10.4 mg/dl
AST	44 Units/L	8-34 Units/L
ALT	31 Units/L	10-49 Unis/L
ALP	99 Units/L	46-116 Units/L
Serum glucose	130 mg/dl	74 – 106mg/dl
Serum lipase	43 Unit/L	12 – 53 Unit/L

A thorough review of previous records were done which confirmed multiple emergency room visits for similar symptoms. On prior presentations, labs were mainly significant for mild leucocytosis and yponatremia, however no source of infection was identified as urinarylsis, urine cultures, blood cultures and stool microbiolocial testing did not reveal and infectious etiology for the patient's symtoms, and leucocytosis resolved within 24 hours of fluid hydration. Liver enzymes tests, serum lipase levels and serum alcohol levels had also been within normal limits. Chest, renal, abdominal and pelvic imaging with radiographs, ultrasound and CT scans did not reveal and anatomical cause for vomting. On his most recent prior presentation, he was given a referal for gastroenterology follow-up for possible endocsopy, however, patient re-presented prior to his appointment. One consistent finding on each prior admission was a postive urine drug analaysis for cannabis and no other drugs identified. He had been refered to gastroenterology for outpatient endoscopy on his last presentation, but did not follow up.

On this presentation, he was commenced on intravenous fluid hydration and antiemetic therapy with ondansetron, metoclopramide and pantoprazole with clinical improvement and normalization of renal function over the following two days. He was counselled extensively on cannabis cessation and was contemplative. He was subsequently discharged.

## Discussion

CHS is a clinical syndrome characterized by recurrent episodes of nausea and vomiting which is associated with chronic use of cannabis [4]. Most patients often report improvement of symptoms with hot water baths and complete resolution of symptoms with cannabis cessation [2,4].

The exact mechanism resulting in CHS is uncertain. Cannabis interacts with many endocrine, gastrointestinal and neurologic receptors and has been noted to improve and promote emesis [6]. The anti-emetic properties of cannabis are thought to be related to the binding of cannabinoids to CB1 receptors and 5-HT3 receptors in the dorsal ventral complex. The proposed pathophysiology cannabinoid hyperemesis involves chronic overstimulation of 5-HT3 receptors in the area postrema of the dorsal ventral complex by cannabinoids [7,8].

Another proposed mechanism for the pathophysiology of CHS is the activation of the transient receptor potential vanilloid subtype 1 (TRPV1) receptor by cannabinoids. These receptors are also found in the area postrema of the medulla oblongata along with the vagus nerve gastric enteral nerves where they play a role in gastrointestinal motility and central causes of nausea. It is hypothesized that chronic activation of these TRPV1 receptors by cannabinoids results in changes in gastric motility, leading to abdominal pain and promotion of vomiting as well as central nausea. TRPV1 receptors are also found in the skin and it is thought that cutaneous stimulation of these receptors, usually by heat, temporarily counteracts the changes in gastric motility caused by cannabinoid stimulation, hence explaining the improvement of symptoms of CHS with hot water baths [9].

Chronic long-term use of cannabis has been highlighted as the major risk factor for CHS, however, the exact duration or quantity of cannabis use precipitating this syndrome has not yet been determined [10]. Daily cannabis use is thought to increase the risk of CHS more so than the amount of cannabis used [11].

The clinical presentation of CHS is divided into three clinical phases: a prodromal phase, a hyperemic phase and a recovery phase. The prodromal phase is often characterized by nausea and abdominal pain and may last for an extensive period, often months and possibly years. Patients often report a fear of vomiting. The hyper-emetic phase is characterized by recurrent episodes of severe nausea and vomiting which can last 24 to 48 hours and may result in dehydration, acute kidney injury (AKI) and electrolyte abnormalities secondary to dehydration. The recovery phase occurs after the nausea and vomiting resolve and may coincide with the cessation of cannabis. The duration of the recovery phase is variable, and may last days to months [11,12].

Similar to the case presented here, patients reported in the literature present in the hyper-emetic phase and usually have a history of many healthcare visits and hospitalizations for severe nausea and vomiting in which a complete laboratory and imaging workup often reveals no precipitating cause [13].

Our patient was noted to have significant renal impairment on this presentation, with lab investigations pointing to an AKI with FeNA of 0.2%. His acute kidney injury was most likely due to dehydration from gastrointestinal losses (mainly vomiting) and decreased oral intake during the episode of nausea and vomiting, and resolved with intravenous fluid hydration.

One other consideration for his AKI is cannabinoid intoxication. Some cases in the literature have made an association between the use of synthetic cannabinoids and the development of AKI, however, there have been no reports of natural cannabis associated with AKI. The mechanism of synthetic cannabinoid-associated AKI remains unclear. Bhanushali et al. first described four cases of acute kidney injury in patients presenting with nausea, vomiting and abdominal pain after using synthetic cannabinoids. In these cases, acute kidney injury occurred in young previously healthy individuals and biopsy-proven acute tubular necrosis was identified as the cause in 75% of these cases [14]. Our patient reports only using natural cannabis and denied any other illicit drug use. His urine drug screen was only positive for cannabis, however, synthetic drugs are often not identified in routine urine drug screens.

The diagnosis of CHS is often made on clinical criteria which include recurrent severe episodes of nausea and vomiting and epigastric or periumbilical pain in a patient with chronic cannabis use, often associated with compulsive hot baths and resolution of symptoms with cessation of cannabis [13]. Symptoms that may support the diagnosis of CHS, if present, include weight loss of more than 5kg, occurrence of symptoms in the early morning, age <50 years old, history of normal bowel habits and negative workup including laboratory and imaging [11].

Many checklists have been proposed to assist with the diagnosis of CHS, with the most recognized being the Rome Criteria IV, however, even this set of criteria has several limitations including no clear outline on the duration or quantity of cannabis use precipitating this condition and the number of episodes of vomiting required to be considered CHS [8,13].

The management of CHS is dependent on the severity of symptoms and complications associated with CHS. It often includes aggressive rehydration intravenous fluids, antiemetic therapy and proton pump inhibitors [15]. Several anti-emetics are used in the management of the hyper-emetic phase of CHS inclusive of 5-HT3 antagonists such as ondansetron and dopamine antagonists such as metoclopramide, however, some cases of CHS are often refractory to these therapies. Given the clinical characteristic of improvement of symptoms with hot baths, topical capsaicin had been proposed to be a therapeutic option. Topical capsaicin also stimulates the TRPV1 receptors in a similar way to heat, which leads to temporary improvement of the symptoms of CHS [16,17]. Fortunately, in our case, our patient had resolution of his symptoms with ondansetron, metoclopramide and the use of proton pump inhibitors.

CHS has a good prognosis provided that the patient commits to cessation.

## Conclusions

CHS is a clinical syndrome associated with chronic long-term cannabis use, though the duration and quantity of cannabis which precipitates the onset of this syndrome have not yet been specified. The recurrent clinical symptoms of severe nausea and vomiting in association with abdominal pain often prompt multiple emergency room visits in patients who suffer from these symptoms and lead to significant morbidity. These patients often undergo extensive workups to rule out other potential causes for these symptoms leading to increased healthcare costs.

CHS is underrecognized as a potential cause of young patients presenting with recurrent cases of acute nausea and vomiting, especially with a history of chronic long-term cannabis use. Our case highlights the need for consideration of CHS as a differential diagnosis in these cases. With increasing prevalence of cannabis worldwide, many clinical complications are becoming evident, including AKI associated with the use of synthetic cannabinoids. But with more reported cases similar to ours, AKI may become apparent as a potential complication of cannabis intoxication by mechanisms other than dehydration.
